# Supplementary material for: Abdominal fat depots and their association with insulin resistance in patients with type 2 diabetes
Source: PLoS One. 2023 Dec 8;18(12):e0295492. doi: 10.1371/journal.pone.0295492 (PMC10707599; doi:10.1371/journal.pone.0295492)
Supplement: Attachment — Submitted filename: Response_to_Reviewers_04_04_2023.docx [file pone.0295492.s003.docx]

**Response to Reviewers’ comment - Manuscript no. PONE-D-22-13467**

Title---*It is the ectopic liver fat, not the abdominal visceral fat that shows association with insulin resistance, Type-2 Diabetes and metabolic syndrome in Asian Indians.*

**Reviewer#1: Major criticisms**

*1. It should be noted that data of HOMA-IR in this study may not be normally distributed (SD is higher than mean) particularly in T2D subjects (Table 1), therefore the analysis of correlations between HOMA-IR and compartmental fat or other variables by Pearson’s method may not be appropriate and may result in misleading information. Data of HOMA-IR should be log-transformed prior to analysis if Pearson’s correlation is used. Please consult statistician for this matter.*

**Response:**

As per comments, log transformation of HOMA-IR has been done and included in analysis in revised manuscript. Although, results are nearly similar.

*2. Given data from Table 1 and Table 2, I suspect that the majority of MetS group has T2D. (How many patients in MetS group had not been diagnosed with diabetes in this study?) If it is so, separating analysis of MetS from Non-MetS may not give you additional information since it will be similar to those of T2D group.*

**Response:**

Out of 67 people with metabolic syndrome, 58 had DM, while only 21 persons with diabetes were in non-metabolic syndrome group (21/97). We agree with your interpretation.

**Minor criticisms**

*1. Given the SAT and VAT measurements are in part operator-dependent, the authors should specify how many radiologists involved in the study. What is the intra- or inter-observer variation of those measurements?*

**Response:**

Only one investigator was involved in SAT and VAT measurements and he was blinded from patient clinical and biochemical data.

*2. Since T2D patients were more obese than control (non-diabetes), it is interesting to see whether the differences of fat-compartment between T2D and control shown in Table 4 was changed after adjustment with BMI.*

**Response:**

That table has now Table 3 in current version. As suggested, we have also included BMI-adjusted P values also for all the groups across all the three adipose sites. We could see that no major change in the overall results, when study data were adjusted to BMI.

*3. The authors may give more discussion regarding the correlations of insulin resistance and fat compartment in the quartile 4 of Table 6 whether it is specific for T2D (since T2D is the majority of population in this Q4) or it can be generalized to non-T2D as well.*

**Response:**

Thanks for advising on this important point. It has been discussed in detail in the discussion section. As it has been discussed that fat re-distribution from SAT to ectopic liver compartment is true for T2D and MetS group, majority of them falls in Q4. This table has now become Table 5 in current version.

*4. The authors should make it clear that the positive association of liver fat with insulin resistance or T2D or MetS are not independent from each other since the latter two of which have insulin resistance as a common factor.*

**Response:**

Thanks for pointing it out. The same has been mentioned in discussion (lines 196-202)

*5. In contrast with other studies, the authors should comment why, in this study, VAT was not associated with insulin resistance even in those with the most severe insulin resistance. It is interesting to note that the VAT area is quite high even in lean non-diabetic population supporting the tendency to accumulate central fat in this population.*

**Response:**

On re-analysis of the data using log HOMA-IR (instead of HOMA-IR), it showed borderline (though statistically insignificant) positive association with the VAT mass, particularly in its highest quartile. Therefore, the whole manuscript has been revised accordingly.

*6. Figures may not be necessary since they do not give more additional information than tables.*

**Response:**

Figures are now removed as per comment.

*7. Reference format should be revised according to the Journal style.*

**Response:**

References format has been revised.

**Reviewer #2:**

**Introduction**

*1. The second and third sentences refer to IR in the pathophysiology of T2D and MS. IR is a key feature in the pathophysiology of T2D. However, since metabolic syndrome is not a disease per se but rather a cluster of various risk factors of which IR is one (but not in always, depending on the criteria that were used), I would suggest the authors to rephrase these sentences.*

**Response:**

Sentence has been rephrased (line 41).

*2.Based on the objectives, the associations with MS is a secondary objective. I would therefore suggest the authors to first focus on T2D in the introduction and then mention MS. Also, the authors should clarify the rationale for assessing the associations of central and ectopic liver fat in those with MS. This is not clear in the introduction.*

**Response:**

We agreed that discussion and data focused to T2D. Central (non-ectopic fat i.e., VAT, and liver fat) were different in occurrence and distribution and different stage of patho-biogenesis; hence discussed.

*3.Sentence starting on line 10-11: Please include the comparison group. The author stated that...” they are more insulin resistant and have a higher prevalence of T2D and MS”. More than “who”? The reference for this sentence was given as Raji et al. 2001. Please cite more recent studies or even a systematic review if available.????*

**Response:**

Systematic reviews are not available to best of authors knowledge. Please let us know if any.

*4.Last paragraph regarding the objectives of the study: The objectives need to be clearer. The authors need to clarify whether the first objective stated as “to find the relationship between MRI assessed abdominal fat distribution … and IR” and the second objective stated as “…to assess the relationship in different quartiles of IR in these subject” refers to those with and without type 2 diabetes.*

**Response:**

Corrected as per your comment.

**Methodology**

*5. General comment – it is not clear whether medical records were used to identify the participants retrospectively or whether participants were included prospectively during visits at the hospital and whether all the participants (T2D and non T2D, MS and non-MS) received OGTTs. Can the authors please clarify this?*

**Response:**

Study subjects’ medical record along with history, were used to identify participants and those not having T2D confirmed with OGTT as mentioned.

*6. Please elaborate whether the participants were recruited from one particular department eg. diabetes clinic or from different departments.*

**Response:**

Participants were recruited from Single department.

7. Line 6 in this paragraph: omit the word “normal” and just mention “individuals without T2D”.

**Response:**

Corrected as per suggestion.

*8. These individuals without T2D, were they tested for T2D and tests were negative or were they never tested. If the latter is the case, this group may contain undiagnosed T2D individuals. This should be considered in the interpretation of the findings and as a limitation.*

**Response:**

Those without T2D were tested with OGTT to confirm status as mentioned in methodology.

*9. Details regarding how participants with MS were selected and the criteria used should be moved from the clinical and biochemical assessment section to this section. A reference should be included for the IDF definition of MS. The rationale for choosing the IDF criteria should be provided. Clinical & Biochemical assessment.*

**Response:**

IDF harmonizing criteria was used as it has specific consideration to our population (South East Asia). Reference mentioned.

*10. In line 13 in the section it is mentioned that HOMA-Beta was also calculated, however, this was not shown in any of the descriptive tables. HOMA-Beta is another key factor in the pathogenesis of T2D, together with IR. In my opinion it may even be more important than IR. Even though IR may be present, hyperglycemia will not occur as long as the beta cell function remains optimal and can compensate for the level of IR. Also, a recent review published in Diabetologia (Venkat Narayan 2020) suggested that South Asians may have reduced insulin secretory ability. It would therefore add to this paper to explore the associations of central and ectopic fat depots with B-cell function.*

**Response:**

South Asian were traditionally considered to have reduced secretory capacity but recent clusters studies have shown different phenotypes ranging from insulin resistance to insulin deficient and combined insulin resistance and deficiency. As the aim of study was to explore insulin resistance rather than Diabetes pathogenesis; HOMA-beta analysis not taken into analysis in this study.

**Radiological investigations**

*11. The method used for the quantification of VAT, SAT and ectopic liver fat – requires more detail. The MRI procedure that was used eg. Dixon needs to be stated. I assume the area of SAT and VAT were done on 1 slice of 3 mm thickness. This needs to be clear. How was the ROIs drawn in the liver – automatic, semi-automatic or manual? Please include the precision of the methods that were used.*

**Response:**

Dixon method used by single radiologist with ROI drawn manually.

**Statistical analysis**

*12. Please include the statistical software used for the analysis.*

**Response:**

SPSS26 was software used for the analysis.

*13. The authors should provide more detail regarding the distribution of the key variables used in the correlation analysis and whether any transformation of non-normally distributed variables was done prior to the Pearson correlation analysis and ANOVA (please see comment below).*

**Response:**

Done

*14. An ANOVA was done in Table 3 but this is not mentioned in the statistical analysis. Also, I would advise a post-hoc test to be done to determine which quartiles were significantly different from each other.*

**Response:**

Done

*15. I would suggest that the authors use a small caps “r” to abbreviate the correlation coefficient.*

**Response:**

Done

**Results**

**General characteristic**

*16. It is stated in the 3rd line that both groups were matched for age and sex. However, the male to female ratio is given for the whole study population. I would suggest that the authors also indicate the proportion of males and females per group (T2D vs nonT2D), either in the text or in Tables 1 and 2.*

**Response:**

Done

*17. Since this study used the IDF definition of MS. This means that having hyperglycemia or T2D is not a compulsory criterion. Besides comparing the MS and non-MS groups it will add to this paper to also mention the proportion of people classified as MS that have diabetes and hyperglycemia (fasting glucose > 100mg/dL).*

**Response:**

58 out of 67 people with MS were having T2D and out of 91 people without MS 21 were suffering from T2D.

*18. A visual presentation of the association between HOMA-IR with VAT, SAT and liver fat as continuous variables, for the whole group, by T2D status and by MS status, would be better. Chart 1 is just a repeat of Table 5. I would suggest showing the graphs with continuous variables and the correlation coefficient and p-value indicated on the graph. This will replace the current Table 5 and Chart 1.*

**Response:**

Done

*19. Graph 1: “Correlation of HOMA-IR to fat depot on each HOMA IR Quartiles and Chart 3 as well as Table 6 provide the same information. I would suggest keeping the table and to omit the 2 graphs.*

**Response:**

Done

*20. Table 3 and 6: Do these tables include all subjects (n=160) or only those with diabetes (n=80)? I suggest that the authors clarify this in the title of the table and include the number of participants overall and in each quartile.*

**Response:**

Whole population is included.

**Discussion**

*21. The authors should consider the following as potential reasons for finding no association between VAT and HOMA IR in this study:*

*-	Association may not be linear – a curvilinear association has been described such that at higher VAT levels, no changes in IR occurs – which may explain the lack of association amongst those with higher VAT.*

*-	HOMA-IR reflects liver insulin sensitivity and therefore the close association with hepatic fat is understandable but VAT may be more important for peripheral insulin sensitivity? Can this be a shortcoming of using HOMA-IR instead of measures of whole-body or peripheral insulin sensitivity? This should be discussed.*

*-	Precision of measuring VAT vs ectopic liver fat could this have influenced the results? It is therefore important to state the precision and validity of the methods used to measure the fat fraction in these depots in the methodology section and it can be reiterated in the discussion.*

**Response:**

It is done

**Reviewer #3**

*1. In the 'methodology' part, you can find information: ‘Ectopic liver fat was measured using liver intensity on Osirix software’.*

*There is no information from which sequence/sequences the software displayed the fat content automatically - I think the methodology should be described in more detail.*

**Response:**

Dixon method used on Osirix software by single investigator, and as per your suggestion included in methodology.

*2. 160 people were included in the study, 80 with type 2 diabetes and 80 without diabetes (Table 1). In Table 2, the 'study population' is only 158 people - what happened to the other two people?*

**Response:**

While study population was 160, 2 subjects have data missing on lipid profile hence not included in metabolic syndrome analysis. So, when talking about metabolic syndrome wise population in analysis reduced to 158.

*3. Insulin resistance, i.e. the lack of tissue sensitivity to insulin, is a common metabolic disorder that can lead to the development of type 2 diabetes. As I understand it, the study assessed 80 people with already developed type 2 diabetes, not only insulin resistance, and 80 healthy people. Thus, the paper did not concern the relationship between insulin resistance and the amount of subcutaneous, visceral and ectopic liver fat but the relationship between type 2 diabetes and the quantity of subcutaneous, visceral and ectopic hepatic fat. Please clarify.*

**Response:**

As we also analysed relationship in people without diabetes (confirmed by OGTT), along with diabetics so we concern with IR rather than T2D.

*4. What were the HOMA-IR values for people with insulin resistance - as long as the study included people with insulin resistance solely and not only those with developed diabetes?*

**Response:**

Rebuilt and done

*5. There is no information on how many and what components of the metabolic syndrome (except diabetes) were found in the group of patients with MS (Table 2).*

**Response:**

As we did not stratify according to number of features of MS, we took 3 or more features as per IDF harmonizing criteria for MS.

*6. Table 2: Patients with MS (metabolic syndrome) accounted for 67 of 158 and without MS 91 of 158. So there are also patients with T2D in the 'without MS' column. This is misleading, I think this table should be reworked.*

**Response:**

Yes, 58 out of 67 people with MS were diabetic while 21 out of 97 non-MS people were diabetic. As IDF criteria diabetes is not compulsory hence it is not misleading.

*7. There are no citations and descriptions for charts 1, 2 and 3 in the text.*

**Response:**

Done

*8. Table 4 is not cited in the text of the paper.*

**Response:**

Done

*9. A broader discussion of tables and graphs in the text would improve the readability, comprehensibility, and, thus, the value of the entire work.*

**Response:**

Done

*10. The citation brackets should be standardized - authors use both [] and ()*

**Response:**

Done

*11. There is no abbreviation list.*

**Response:**

Done

*12. References: use 6-8, 10-13, 16-20, not '(6,7,8,10,11,12,13,16,17,18,19,20)'*

**Response:**

Done

**Reviewer #4:**

**Minors:**

*-The small sample size is a limitation, especially regarding quartile analyses. Therefore, it is good to mention it as a caveat and the results to be reported with more caution.*

**Response:**

These limitations of the study are mentioned in the discussion section of the manuscript.

*-In the abstract result section, stating the reciprocal relation between IR and SAT does not seem correct, as this will require bidirectional causal analysis. Perhaps the authors meant an inverse relation!*

**Response:**

Thanks for pointing it out. Appropriate correction has been made.

*-Due to multiple hypotheses testing, control for false discovery rate needs to be done (e.g., reporting FDR corrected p-values).*

**Response:**

We calculated correlations over aggregate sample values not for each individual separately so FDR correction would likely not required.

*-In the introduction, considering MRI and CT as the gold standard is incorrect, and they have their own limitations (e.g., cost and low precision). Even biopsy, which is usually considered the gold standard, has its caveat of being invasive.*

**Response:**

Thanks for pointing it out. Appropriate correction has been made.

*-As the study was conducted during COVID, good to mention whether it did affect the implementation of their research or not. Also, as one of their exclusion criteria was the presence of an infection, were COVID-associated infections recorded? It will be a great addition to their study if they have such registered data. For instance, whether their measurements were recorded before having COVID or after.*

Covid related information was not noted.

*-Spell out the word in the first occurrence (e.g., OGTT).*

*-They have several blood biomarkers measured (e.g., HDL, LDL, VLDL, and TG); why not include their raw value in the analyses?*

*-	In tables, it is good to highlight the significant ones.*

*Thanks for this suggestion. It is done.*

*-Distribution plots are a good addition, and they will help with identifying whether normalization is required or not. Scatter plots by fitting a linear model into it is pretty informative.*

*-A Heatmap plot among all the variables also will make it easier to follow the results.*

**Response:**

Heatmap has now been added.

**Second review**

**Reviewer #1:**

*1. Abstract: The Results section was not yet revised.*

*1.1 The sentence “In T2D and MS, HOMA-IR showed a moderate strong correlation with ectopic liver fat…but not SAT and VAT” is not true. In Table 5, the log-transformed HOMA-IR had no correlation with liver fat in both T2D and MS.*

**Response:**

Thanks for pointing out this important point. The whole data is re-analysed with log HOMA-IR instead of HOMA-IR and corrections are made accordingly throughout the manuscript right from title to inference. The ectopic liver fat still shows significant correlation with Log HOMA-IR. VAT show borderline correlation, which is though statistically not significant, but is marginal only.

*1.2 The next following sentence “On analysing the correlation of abdominal adipose compartment fat mass with IR…., ectopic liver fat shows a positive correlation with HOMA-IR (r=0.52, p < 0.05)” is not correct since the correlation of log-transformed HOMA-IR and liver fat was 0.363 and was not significant (Table 5).*

**Response:**

Among diabetics and MetS group the correlation coefficients are 0.46 and 0.493 respectively and they are statistically significant. As participants in this study were recruited in T2D and non T2D groups separately, therefore analysis of combined group has been removed from analysis.

*1.3 Likewise the sentence “SAT shows an inverse relation with IR with a statistically significant negative correlation in the highest quartile” is also not true given the p values of 0.05 in log-transformed HOMA-IR and SAT analysis (Table 6)??*

**Response:**

It is *P* < 0.05 and has been corrected.

*1.4 The Conclusion should be revised in accordance with the new results*

**Response:**

The conclusions have been revised accordingly.

**2. Methodology**

*2.1 The method of statistical work which include the log-transformed of HOMA-IR data prior to Pearson’s correlation analysis and ANOVA (Table 3) should be mentioned in methodology section??*

*2.2 The precision of manually-measured SAT, VAT area by the radiologist for example intra-observer CV of measurements should be reported*

**Response:**

Fat analysis was done by a single person.

**3. Results**

*The correlation results (r) and their significance level (p) between HOMA-IR and fat compartments were changed after log-transformed data of HOMA-IR was applied. Therefore, the r and p values should be revised accordingly*

**Response:**

The values have been revised accordingly

**4. Discussion**

*4.1 The statements “…in its highest quartile, HOMA-IR showed a significantly negative correlation with SAT” and the following “SAT fat mass as a protective role in the pathogenesis of IR” of which it appeared in several occasions in the Discussion and Conclusion was not quite right since the p value was 0.05. It is safer if the authors rephrase the statements “…HOMA-IR tended to show a negative correlation with SAT” and “SAT may have a protective role…”*

*4.2 In the second paragraph of Discussion, the authors quoted the significant correlation (r) results of HOMA-IR and liver fat in the whole (r=0.52) T2D (r=0.609) and MS (r=0.673) population, of which they are not correct values. In log-transformed HOMA-IR data, those were 0.363, 0.46 and 0.493 respectively (Table 5). All are not significant.*

**Response:**

The log transformed HOMA-IR are used in correlation analysis and accordingly title, results and discussion and conclusions are modified.

*4.3 The authors should add a little more discussion why VAT was not correlated with HOMA-IR in this study of which it is in contrast with several others. It should be noted that log-transformed HOMA-IR and VAT tended to be correlated at the highest quartile of IR (p=0.078)*

This is a very important and the whole manuscript has now been revised accordingly. Thanks for pointing it out. This inconsistent finding is discussed in the discussion section lines 248-253.

**5. Tables and Figures**

*5.1 In Table 5 and 6 regarding the correlation of HOMA-IR and fat depots, only the log-transformed HOMA-IR data should be reported.*

**Response:**

Only log transformed HOMA-IR has been reported in the table.

*5.2 Figure1 is not necessary, it repeated what appeared in Table 6.*

**Response:**

The figure has been removed.

**Reviewer #4:**

*The authors have not put enough effort into addressing the comments. Small sample size, multiple hypothesis testing, and not correcting for that are important caveats that need to be at least discussed and acknowledged. Visualizing the distributions of the data or a correlation matrix/Heatmap plot among the variables would greatly help understand the data and interpret the results.??*

**Response:**

Thanks for the comments. The manuscript has now been thoroughly revised and previous suggestions of reviewers that were not addressed in the second revision are now incorporated in this third revision. The correlation matrix is added as figure 1 in the results. The limitations mentioned above are incorporated in the discussion (lines 256-60)

We have made substantial improvements as per comments of our revered reviewers and hope the current version of our manuscript would come up to the expectation of them. We also enclosed a correlation plot to feature relationships across variable of interest.
