# Supplementary material for: Abdominal fat depots and their association with insulin resistance in patients with type 2 diabetes
Source: PLoS One. 2023 Dec 8;18(12):e0295492. doi: 10.1371/journal.pone.0295492 (PMC10707599; doi:10.1371/journal.pone.0295492)
Supplement: Attachment — Submitted filename: Response_to_Reviewers_22_06_2023.docx [file pone.0295492.s004.docx]

**Response to Reviewer’s comment - Manuscript no. PONE-D-22-13467**

**It is the Fatty Liver, Not the Adipose Depots Surrounding the Intraperitoneal Organs That Shows Association with Insulin Resistance in Asian Indian having T2DM & Metabolic Syndrome.**

**Editor’s comment:**

*Put together, the title, methodology and conclusion do not clearly tie.*

*Visceral adipose tissue (VAT) refers to the fat that surrounds the body's internal organs, particularly those in the abdominal region such as the liver, pancreas, and intestines also including omental fat. It's one of the two main types of abdominal fat, with the other being subcutaneous adipose tissue which lies just underneath the skin. This means that ectopic liver fat could also be part of VAT. As per methodological description, what was described as VAT was literally omental fat, none of the other depots that makes up VAT were measured (or were measured but not presented and described). Based on that the title and conclusion of this study do not reflect what was exactly done. The authors needed to include the other depots making up VAT for them to conclude that it is only the ectopic liver fat and not other depots that are associated with insulin resistance. Additionally, the authors should directly say omental fat rather than VAT, if what was measured and referred to as VAT was only omental fat. Furthermore, what is described as subcutaneous fat was only measured around the abdomen. Using SAT is very confusing given that only abdominal SAT was measured. The authors need to be very specific.*

*It is also important for the authors to be very clear in the use of terms such as ectopic fat. Ectopic fat may refer to the deposition of triglycerides within cells of non-adipose tissue that normally contain only small amounts of fat. These tissues include the liver (resulting in fatty liver disease), muscles, heart, and pancreas. Ectopic fat accumulation is believed to occur when the storage capacity of healthy adipose tissue (like subcutaneous fat) is exceeded, leading to an overflow of excess free fatty acids into the circulation and uptake into these other tissues.*

*Therefore, while visceral fat is a type of adipose tissue located in a specific area (around the internal organs in the abdominal cavity), ectopic fat refers more to a pathophysiological condition where fat is stored in cells of tissues that usually do not store large amounts of fat. Based on this fact, it might be that what is associated with insulin resistance is FATTY LIVER and not what they described as ectopic liver fat. Reflecting on these confusions the authors need to clearly elaborate and justify the use of terms they decide to use with much clarity.*

**Response**
Thanks for pointing out this terminology confusion. We have now clarified this confusion and have selected new terms for the three abdominal fat / adipose compartments measured , i.e. liver fat, subcutaneous adipose tissue (SAT) and adipose tissue compartment surrounding intraperitoneal organs (S-VAT). These terms are selected in such a way that they reflect exactly what was measured in this study. It is mentioned in the methods section (line 124-138). The title, methodology and conclusions are changed accordingly.

Reviewer #1:

1. Abstract

**Comment**

*Results: line 33-35; please correct the r and p values of the correlation between SAT and IR in highest quartile. It should be r = -0.2843 and p = 0.05*

**Response**

It has now been corrected.

2. Methodology

**Comment**

*Analysis of variance for between groups comparison should be included in the statistical analysis*

**Response**

In this study, we used student T-test for parametric variables which is considered to less likely commit an error in comparison to ANOVA. Moreover we always compare two groups at a time so ANOVA was not essential*.*

3. Results

**Comment**

*3.1 line 174-176: the r-value of the correlation between HOMA-IR and ectopic liver fat in subjects with MetS was 0.673, of which it was in disagreement with that of 0.493 as shown in Table 3. Which number is the correct one? It should be noted that the r-value of 0.493 was not significant by the previous version of the manuscript (Table 5). The p-value of the correlation was also in disagreement with that shown in Table 3.*

**Response**

Table 5 and 6 in the first version correspond to table 3 and 4 in the second version. Earlier table 5 shows correlations of HOMA-IR with different fat mass whereas in second version, table 3 shows correlation of log-HOMA-IR with BMI-adjusted fat masses. That’s why both *r* and *p* values are different (*r* = 0.493, *P* < 0.0000001) than the previous one (*r* = 0.673, *P* < 0.05). We have also updated these values between lines 181-183 in text.

**Comment**

*3.2 Likewise, the r-value of the correlation between HOMA-IR and VAT was incorrect.*

**Response**

Table 5 and 6 in the first version correspond to table 3 and 4 in the second version.

**Comment**

*3.3 Please correct the symbol used to indicate the p-values in Table 3, for examples p = 0.97 not p <0.97*

**Response**

Corrected

**Comment**

*3.4 Line 181: the comparisons of subject characteristics according to IR quintiles was in fact, shown in Table 5 not Table 4. Likewise, in line 188, the correlation of fat compartments with IR quintiles was shown in Table 4, not Table 5*

**Response**

Corrected

**Comment**

*3.5 Line 191-193: the statement “SAT showed statistically significant negative correlation with log HOMA-IR in the highest quintile of IR” is over-claimed since the p-value was not significant (p=0.05) as shown in Table 4*

**Response**

This sentence has now been updated as:

“SAT showed an almost statistically significant negative correlation with log HOMA-IR in the highest quartile of IR (r = -0.2843 P ≈ 0.05 in quartile IV)”.

**Comment**

*4. Discussions and Conclusions*

*It appears that the authors over-claimed SAT as a protective factor against IR in several occasions, for examples in line 207-209, 247-248, 266-268 although it was not supported by the study results.*

**Response**

Thanks for pointing out this important issue. The discussion and conclusions are re-written accordingly.
