# Supplementary material for: Abdominal fat depots and their association with insulin resistance in patients with type 2 diabetes
Source: PLoS One. 2023 Dec 8;18(12):e0295492. doi: 10.1371/journal.pone.0295492 (PMC10707599; doi:10.1371/journal.pone.0295492)
Supplement: Attachment — Submitted filename: Response_to_Reviewers_27_10_2023.docx [file pone.0295492.s005.docx]

**Respected Dr Mashili,**

Thank you very much for conducting the 3^rd^ round of review for our submitted manuscript. We have gone through the comments and suggestions given by our revered Reviewers and could come up with our revised manuscript which hopefully satisfy their concerns.

A point-by-point comments is enclosed herewith:

**Comments to the Author**

4. Have the authors made all data underlying the findings in their manuscript fully available?

Reviewer #5: No

**Response**

*All data underlying the findings in manuscript has now been fully available in Supplementary File.*

6. Review Comments to the Author

Reviewer #5: I have reviewed the manuscript, the following minor issues needs to be addressed.
1. P-values should be reported with uniform decimal places throughout the manuscript also there should be a consinstence in writting letter "p". To enhence readability there should be no mixing of small and capital letter

2. Authors should correct all typographical errors throughout the manuscript. For example, line 141. its written p-val

**Response**

*P values have been reported with uniform two-decimal places and also following consistent writing style and Typographical errors have been rectified to best of our knowledge.*

3. Authors should consider providing interpretation of all abbreviations when they are used for the first time in the text, for example line 118. "IDF" there is subsequent definition of the abbreviation in parentheses

**Response**

*Resolved*

4. reference no 22 is incomplete.

**Response**

*In is now complete*

5. I suggest authors considering to use non-parametric correlation analysis, to see what direction of relationship between liver fat and IR they get. this is because there is possibility of log transformation changing the directions of correlation relationship besides the fact that it transforms non-normally distributed data into a more normal distribution.

**Response**

No change in directionality was observed when we used chi-square test over log-transformed values.

Reviewer #6: While you have tried to address all the comments, the comments from the editor haven’t been sufficiently addressed. There is still some confusion between the conclusion made and what exactly was measured. You conclude that it is the liver fat that shows association and not visceral adipose tissue, whereas the visceral adipose tissue also include liver fat. Additionally, you also include metabolic syndrome in your title, discussion and conclusion while the methodology doesn’t describe or show how patients with metabolic syndrome were selected and recruited. Furthermore, your title is too conclusive while not clearly supported by data. You would rather have written “Abdominal fat depots and their association with insulin resistance in patients with type 2 diabetes” and have the freedom to describe your findings rather than having a very conclusive title. The small sample size is also hindrance for such a conclusive title.

**Response**

*Following updates have been made to resolve the concerns of reviewers:*

1. *The title of manuscript has been changed to ‘Abdominal fat depots and their association with insulin resistance in patients with type 2 diabetes’ instead of earlier conclusive title and also justifying the low sample size.*
2. *Methodology to assign Metabolic Syndrome to individual has been included in Material and Method section.*
3. *The description and terms used for fat depots are transformed to common terms*
4. *In-place of visceral fat, we used term ‘intra-peritoneal visceral adipose tissue fat mass surrounding the vital organs (IPAT-SV)’ to discriminate it from liver fat*
